# The ATM Gene in Breast Cancer: Its Relevance in Clinical Practice

**DOI:** 10.3390/genes12050727

**Published:** 2021-05-13

**Authors:** Luigia Stefania Stucci, Valeria Internò, Marco Tucci, Martina Perrone, Francesco Mannavola, Raffaele Palmirotta, Camillo Porta

**Affiliations:** 1 Division of Medical Oncology, Department of Biomedical Sciences and Human Oncology, University of Bari ‘Aldo Moro’, A.O.U. Consorziale Policlinico di Bari, 70121 Bari, Italy; valeria.interno@libero.it (V.I.); marco.tucci@uniba.it (M.T.); martina.perrone91@hotmail.com (M.P.); francesco.mannavola@gmail.com (F.M.); camillo.porta@gmail.com (C.P.); 2 National Cancer Research Center, Tumori Institute IRCCS Giovanni Paolo II, 70121 Bari, Italy; 3 Interdisciplinary Department of Medicine, Section of Sciences and Technologies of Laboratory Medicine, University of Bari, 70121 Bari, Italy; raffaele.palmirotta@uniba.it

**Keywords:** *ATM* gene, breast cancer, risk, outcome, treatment

## Abstract

Molecular alterations of the Ataxia-telangiectasia (*AT*) gene are frequently detected in breast cancer (BC), with an incidence ranging up to 40%. The mutated form, the Ataxia-telangiectasia mutated (*ATM*) gene, is involved in cell cycle control, apoptosis, oxidative stress, and telomere maintenance, and its role as a risk factor for cancer development is well established. Recent studies have confirmed that some variants of *ATM* are associated with an increased risk of BC development and a worse prognosis. Thus, many patients harboring *ATM* mutations develop intermediate- and high-grade disease, and there is a higher rate of lymph node metastatic involvement. The evidence concerning a correlation of ATM gene mutations and the efficacy of therapeutic strategies in BC management are controversial. In fact, *ATM* mutations may sensitize cancer cells to platinum-derived drugs, as BRCA1/2 mutations do, whereas their implications in objective responses to hormonal therapy or target-based agents are not well defined. Herein, we conducted a review of the role of *ATM* gene mutations in BC development, prognosis, and different treatment strategies.

## 1. Introduction

The Ataxia-telangiectasia mutated (*ATM)* gene is an oncosuppressor, located on chromosome 11q23, that encodes a 350-KDa protein consisting of 3056 amino acids [1]. It belongs to the superfamily of phosphatidylinositol 3-kinase-related protein kinases (*PIKKs*). The *PIKK* superfamily includes six serine/threonine kinases showing a sequence similarity to phosphatidylinositol 3-kinases (*PI3Ks*), including *ATR* (*ATM*- and *RAD3*-related), DNA-PKcs (DNA-dependent protein kinase catalytic subunit), and *mTOR* (mammalian target of rapamycin). The ATM protein is involved in DNA repair and activates DNA damage response pathways [2]; indeed, upon DNA damage, it is recruited to double-strand breaks where it holds the two ends together. *ATM* mutations cause Ataxia-telangiectasia (AT), an autosomal recessive neurodegenerative disorder characterized by a progressive neuromotor dysfunction resulting from several neuropathological processes dominated by gradual cerebellar cortical atrophy, telangiectasia in the eyes and sometimes on the facial skin, thymic degeneration, immune deficiency, recurrent sinopulmonary infections (at least in some patients), retarded somatic growth, premature aging, gonadal dysgenesis, predisposition to lymphoreticular malignancies, and acute sensitivity to ionizing radiation [3,4,5].

In general, the *ATM* gene is involved in cell cycle control, apoptosis, gene regulation, oxidative stress, and telomere maintenance and is deregulated in many malignancies such as breast cancer (BC) [6]. Many *ATM* mutations have been described and associated with a moderate risk of BC development [7]. Furthermore, epidemiological studies based on relatives affected by both AT and BC suggested that heterozygous carriers of *ATM* mutations have a two- to thirteen-fold increased risk of BC development, with a higher relative risk under 50 years old [8,9,10,11].

Previous studies emphasized the evidence of a strong association between *ATM* variants and the risk of BC development. The V2424G variant confers the highest risk of BC development, while the D1853V, L546, and S707P isoforms are associated with the lowest risk [12]. Moreover, next generation sequencing (NGS) analysis revealed that *ATM* is among the most aberrant gene in sporadic cancer (as shown by the COSMIC database), and that loss of heterozygosity in the region of the *ATM* has been detected in approximately 40% of human sporadic BC [13,14,15].

In wider large-scale studies including solid cancers, 5% of patients showed *ATM* aberrations (either mutation or loss). As described, 8% of lung cancer patients showed *ATM* mutations that were largely mutually exclusive with those of *TP53*. More recently, *ATM* alterations have been found in colorectal cancer (CRC) both in patients bearing both microsatellite stable and unstable tumors. In prostate cancer, targeted next-generation sequencing has revealed an 8% incidence of *ATM* mutations. In a range from 1% to 5%, *ATM* mutations were reported in endometrial, kidney, liver, esophageal, ovarian, salivary gland, gastric, thyroid, and urinary tract cancers [12].

Clinical and pathologic characteristics of *ATM*-associated BC have not been well defined, but it is known that *ATM*-mutated BCs are mostly endocrine-positive, dedifferentiated, and more aggressive, and thus have poor prognosis [11].

As regards therapeutic implications, *ATM* aberrations may sensitize cancer cells to platinum-derived drugs, similarly to the effect of *BRCA1* mutations, but have a worse effect in case of radiotherapy (RT). In fact, *ATM* mutations increase the risk for development of a second tumor after RT [16,17,18,19].

## 2. The ATM Gene and Its Role in Cancer

**Figure 1:**
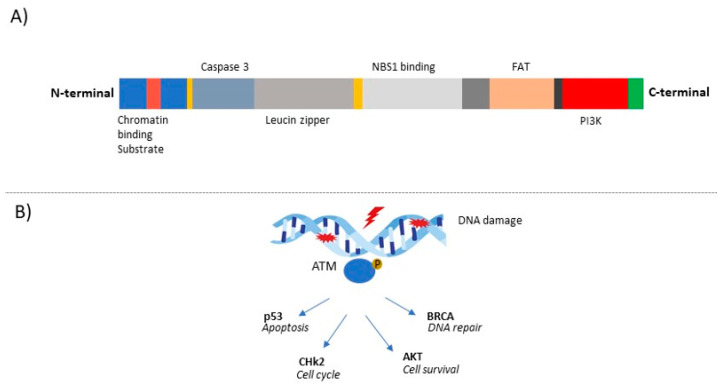
Structure of human *ATM* and function in DNA damage repair. (**A**) ATM is characterized by an N-terminal and a C-terminal half that shows homology to other phosphoinositide-3 kinase (PI3K)-like kinases and a portion that contains a FAT domain (named after the FRAP, ATR, and TRRAP proteins). The N-terminal portion interacts with substrates and cofactors such as *NBS1*, *p53,* and *BRCA1*. In addition, the N-terminal portion is characterized by a proposed chromatin- interaction domain, a nuclear localisation sequence (NLS), two caspase-3 cleavage sites, and a putative leucine zipper region. (**B**) Following DNA damage, *ATM* is recruited and is catalytically activated by autophosphorylation. Once activated, *ATM* serves as a transducer, phosphorylates, and activates other protein kinases such as checkpoint kinase 2 (*CHK2*), which in turn modulates its own substrates, resulting in cell cycle arrest, or *ATM* can also activate *p53*. *ATM* can activate mechanisms involved in DSB repair, such as *BRCA* proteins.

Since the *ATM* protein plays an important role in DNA repair through the activation of enzymes that fix the broken strands, its biology is of interest in cancer research [20]. The physiological structure of the *ATM* protein is characterized by an N-terminal half that is largely unique and a C-terminal half showing homology with other *PI3K*-like kinases such as *ATR*, *mTOR,* and DNA-PKcs. *ATM* contains at least five autophosphorylation sites. The N-terminal portion interacts with substrates and cofactors such as *NBS1*, *p53*, *BRCA1*, *LKB1,* and *BLM* [12]. Once activated, *ATM* phosphorylates many downstream effectors, as illustrated in Figure 1.

Pathogenic variants in *ATM* are common. In particular, around 0.35% of people carry an *ATM* mutation, and there is a strong association between mutations in *ATM* and cancers. Some researchers found a four-fold increased risk for pancreatic cancer, a three-fold increase for stomach cancer, and a two- to three-fold increase for prostate cancer and confirmed the previously known two-fold invasive ductal BC in patients with an *ATM* mutation. They also found a low to moderate increase in risk for male breast cancer, ovarian cancer, colorectal cancer, and melanoma. *ATM* is a large gene with many thousands of locations where a mutation can occur. One common mutation, known as c.7271T>G, is associated with a significantly higher risk of BC (about four-fold) than other *ATM* mutations [21].

In BC, the physiological function of *ATM* is downregulated by malignant cells. In fact, *ATM* phosphorylates *DBC1* (deleted in BC) and promotes apoptosis by the activation of p53 and caspase-2 [22,23,24,25]. Moreover, *ATM* signaling can also be upregulated in cancer cells that have already evaded cell apoptosis through other mechanisms, as seen in melanoma through upregulation of melanoma-associated antigen-encoding (*MAGE*) genes as well as in prostate cancer by activation of the Androgen receptor (AR) [1,2]. Regarding the role of *ATM* mutations on the efficacy of therapeutic strategies, it is well known that activation of the *ATM*-dependent pathway in tumor cells can promote chemoresistance and radiotherapy resistance through the activation of p38 *MAPK* and enzyme transglutaminase 2 [3,26], as well as by inducing enzymes involved in DNA double-strand break (DSB) repair [27]. Although *ATM* is considered as an oncosuppressor gene owing to its role in enhancing chemo-radioresistance of tumor cells, this aberrant protein could potentially be explored as a target for cancer treatment. Moreover, *ATM* signaling induces tumor progression via the NF-kb-dependent pathway that promotes the release of pro-tumorigenic cytokines, as well as the epithelial–mesenchymal transition [28]. Furthermore, in some tumors, *ATM* signaling upregulates the alpha_v_beta_3_ integrin pathway [29], leading to tumor progression and downregulation of immune-mediated cell responses [30]. The tumor-specific alpha_v_beta_3_ integrin expression targeted dendritic cells, facilitating their ability to phagocytose viable therapy-resistant tumor cells, and thereby impaired their ability to cross-prime antigen-specific T lymphocyte, and it has been clearly demonstrated that the integrin plays a critical role in triggering invasive and metastatic activities of tumor cells.

It is well known that *ATM* germline mutations have different effects on tumor cells [31]. Particularly, the increased incidence of BC in families harboring AT has been clearly demonstrated [13], but roles for specific mutations of *ATM* are now emerging. Heterozygous *ATM* mutations are associated with a five-fold higher risk of BC in subjects under 50 years of age [32] and are well classified in the COSMIC (Catalogue of Somatic Mutations in Cancer) database [33]. In addition to conventional BC subtypes, *ATM* heterozygous mutations were recently associated to the predisposition of familial ductal pancreatic adenocarcinoma [34]. *ATM*-silencing mutations or deletions have also been found in other types of tumors, such as lung adenocarcinoma and colon cancer [35,36]. Moreover, *ATM* inactivating mutations are more frequently described in solid tumors with areas of hypoxia, such as gliomas, thus contributing to radiotherapy resistance [37].

## 3. Role of ATM Gene Mutations in BC Susceptibility and Prognosis

*ATM* mutations increase the BC risk, as demonstrated in a recent systematic review and meta-analysis [17]. It is well known that *BRCA1/2* mutations have been associated to a hereditary BC in 5% of patients, but also the incidence of BC in AT families was found to be increased two- to five-fold [12,13,38,39,40,41,42]. The data collected by Moslemi et al. confirmed that *ATM* missense variants increase the risk of BC. The risk of BC is enhanced to a degree ranging from 2.8 to 3.04 [43,44]. Among the different variants explored, the V2424G (c. 7271 T>G) missense variant had the highest association with BC incidence in all subgroups [10,43,44,45,46,47]. While the *ATM* V2424G variant was one of the forms associated with an increased risk of cancer, the *ATM* D1853V missense variant has the least association with BC risk [48]. Moreover, a high association of *ATM* variants with BC is more frequently described in Asian than Caucasian patients, mostly due to racial differences, environmental conditions, or lifestyle. Recently, the Breast Cancer Association Consortium designed a panel of 34 putative susceptibility genes which were checked in 60,466 specimens from BC patients and 53,461 controls. It was demonstrated that protein-truncating variants in five genes (*ATM*, *BRCA1*, *BRCA2*, *CHEK2*, and *PALB2*) were associated with a risk of BC overall (*p* < 0.0001). For protein-truncating variants in *ATM* and *CHEK2*, the odds ratio was higher for estrogen receptor (ER)-positive than ER-negative disease. Rare missense variants in *ATM*, *CHEK2*, and *TP53* were associated with an overall risk of BC (*p* < 0.001). The results of this study defined those genes that could most usefully be included in screening panels predictive of BC. Furthermore, *ATM* proved to be potentially useful for genetic counseling [49]. As previously mentioned, *ATM* mutations also occur in sporadic BC leading to *ATM* gene inactivation, but the mechanisms are still unclear. Different mutations have been described, such as allelic loss [50] and *ATM* epigenetic silencing mediated by CpG island methylation [51]. Post-transcriptional *ATM* regulation mediated by microRNAs has been reported in gliomas and BC [52,53,54]. The miR-18 was reported as a putative *ATM* regulatory miRNA in BC [23], but other studies showed no correlation with *ATM* transcript and miR-18a. A recent study integrated genomic, transcriptomic, and proteomic analyses in a large series of BC and identified tumor subtypes with different subsets of genetic and epigenetic abnormalities [55]. For example, *ATM* loss and *MDM2* amplification proved to be more common in aggressive luminal B subtype BC. The clinical impact of *ATM* downregulation in defining the prognosis of BC patients is limited and not yet validated. Lower levels of *ATM* gene products have been discovered in high-grade BC [56,57,58], suggesting association of the *ATM* mutation with more aggressive disease. Analysis of a large cohort of patients with long-term follow-up showed a strong correlation between the absence of *ATM* protein expression and distant metastasis, resulting in a worse outcome of BC patients [11]. Survival analysis revealed that BC patients harboring inactivation of the *ATM* gene had a shorter disease-free survival (DFS) and overall survival (OS). Moreover, a multivariate analysis by Bueno et al. demonstrated that *ATM* is an independent prognostic factor, in association with clinical–pathological factors such as tumor size and lymph node involvement. Other reports described a strong correlation between *ATM* downregulation and poor survival in patients with p53 wild-type tumors [59,60]. Another study explored patients who underwent multigene panel testing (*MGPT*) between 2013–2019, identifying those harboring *ATM* mutations. Heterozygous germline *ATM* mutation carriers had an increased risk of developing cancer of the breast, pancreas, and other organs [61]. Thus, a decreased *ATM* expression is associated with a worse prognosis in BC, suggesting it may be a potential marker of disease outcome. The majority of patients had intermediate- to high-grade, hormone receptor-positive disease and a possibly higher rate of *HER2* positivity and lymph node involvement. It was also reported that *ATM* expression promotes *HER2*-dependent tumorigenicity in vitro and in vivo. Stagni V et al. [62] demonstrated a correlation between *ATM* activation and a reduced time to recurrence in patients diagnosed with invasive *HER2*-positive BC. Moreover, *ATM* was identified as a novel modulator of *HER2* protein integrity through a complex of *HER2* with the chaperone HSP90, therefore preventing *HER2* ubiquitination and degradation. Thus, since downstream activation of *HER2* by *ATM* may modulate the response to therapeutic approaches, it is conceivable that assessing the *ATM* activity status may be useful in the treatment and prognosis of *HER2*-positive tumors [62].

## 4. Therapeutic Implications of *ATM* Gene Mutations in BC

Nowadays, it is well known that tumors with mutations in genes encoding proteins involved in DNA repair may be more sensitive to treatments that induce cytotoxicity by inducing DNA damage or inhibiting DNA repair mechanisms. As *BRCA1*-mutated tumors may be more sensitive to treatments with platinum derivatives and benefit from treatment with inhibitors of *PARP*, similar strategies could also be hypothesized for BC patients harboring *ATM* gene mutations [63]. Moreover, it is recognized that radiosensitivity is a hallmark of AT syndrome. As a consequence, heterozygous *ATM* mutations increase radiotherapy toxicity [64,65], probably due to defective DNA repair and genomic instability in normal tissues. In view of this evidence, adverse events occurring in BC patients during chemotherapy may be increased in those bearing germline *ATM* mutations. Indeed, some reports showed a high risk of myeloid suppression in *ATM* mutated patients compared to wild-type [65]. *ATM* mutations are predicted to result in an increased sensitivity to platinum-based chemotherapy used for BC treatment [66,67]. Similarly, *PARP*-inhibitors could also be more effective in *ATM*-deficient BC tumors, but they have not been specifically evaluated in *ATM*-mutated BC. *PARP*-inhibitors have shown promising results in tumor cells defective in DNA damage repair, in particular DSBs, as *ATM*-mutated tumor cells. Some studies have considered the efficacy of Olaparib in *ATM*-deficient leukemic cells from patients with leukemia [68] or in patients with gastric cancer [69], but no studies in BC tumor cells have yet been performed. The *ATM*-dependent pathway is also involved in resistance to treatment with *CDK4/6* inhibitors, recently introduced in clinical practice for the treatment of advanced estrogen receptor-positive BC [70]. In this regard, a recent study showed that defects in single-strand break repair in luminal BC can drive endocrine therapy resistance and are closely associated with the *ATM-CHK2-CDC25A* pathway. *ATM*, as a DNA damage sensor, activates *CHK2*, which in turn phosphorylates *CDC25A* that could inhibit the phosphorylation of *CDK4/6*. Therefore, the cross talk between the *CDK4/6-Rb* and the *ATM-CHK2-CDC25A* axes is very important [71]. More recently, Haricharan et al. demonstrated that both *ATM* and *CHK2* gene alterations are required to boost the efficacy of endocrine agents in luminal tumors. In fact, the inactivation of either of these negative cell cycle regulators prevents cell cycle arrest upon ER inhibition [72]. To date, *ATM* alone has not been associated with a high incidence of contralateral BC [73], but genetic variants in *ATM* have been demonstrated to play a clinically significant role in radiation-induced contralateral breast cancer. The Women’s Environmental, Cancer, and Radiation Epidemiology Study, an international population-based case–control study, collected patients with contralateral relapse and a cohort of survivors of unilateral BC. Among women who carried *ATM* missense mutations, those who were exposed to radiation had a statistically significantly higher risk of contralateral BC as compared to those with wild-type or subjects who did not undergo radiotherapy carrying the same predicted deleterious missense variant. Thus, the authors concluded that women who carry rare deleterious *ATM* missense variants and have been treated with radiation may have a more elevated risk of developing contralateral BC. This effect proved to be dose-dependent, and the risk of contralateral BC was greater in cases with *ATM* missense variants. The potential mechanism could be associated to the presence of rare missense variants effectively reducing the level of *ATM* activity, increasing the susceptibility to radiation-induced tumorigenesis [19].

## 5. Discussion: How Could the *ATM* Gene Mutation Influence BC Management?

**Figure 2:**
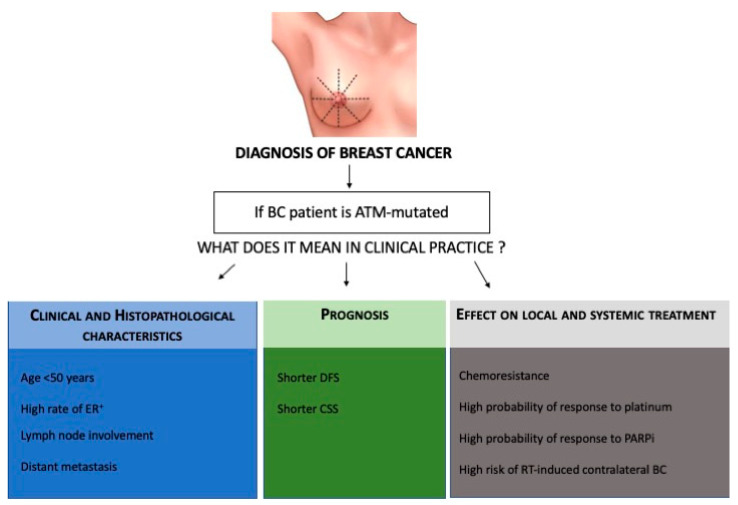
Flow-chart of clinical management of *ATM*-mutated BC patients from diagnosis, treatment to follow-up. BC, breast cancer; ER, estrogen-receptor; DFS, disease-free survival; CSS, cancer-specific survival; *PARP*i, Poly (ADP-Ribose) Polymerase inhibitors.

Traditionally, gene testing or inherited BC genes has focused on women at high risk who have a family history of BC or who were diagnosed at an early age. In a recent study [49], mutations or variants in eight genes as *BRCA1/2*, *PALB2*, *BARD1*, *RAD51C*, *ATM*, and *CHECK2* were found to be significantly associated with BC. To date, clinical practice also relies on the use of gene panel testing of unaffected women with a moderate risk of BC in the family history, in particular, counseling women with *ATM* gene mutations. The management of women with these mutations will consist of screening alone and magnetic resonance imaging (MRI) at the age of 40 years. Nowadays, clinicians are not ready to expand the gene panel test to the general population, and the *ATM* mutation is mostly diagnosed after the diagnosis of BC. Thus, the role of the *ATM* gene in predisposing to BC development seems to be limited. However, the present review aims to guide the clinician to use *ATM* in clinical practice during management of BC (Figure 2).

Firstly, we have learned that *ATM* gene mutations are correlated with specific clinical characteristics of BC such as high risk of ER-positive BC, grade two or three tumors, lymph node involvement, and *HER2*-positivity as well as the development of a contralateral breast tumor in patients resistant to radiotherapy. Thus, *ATM* mutations in BC patients are associated with a worse prognosis. These data could support clinicians in personalizing both treatments, as well as follow-up, in these patients. Moreover, since mutations in *ATM* encoding protein are involved in DNA repair mechanisms, *ATM*-aberrations may also sensitize BC cells to platinum drugs or PARP inhibitors in triple-negative BC, similarly to the effect of *BRCA1* mutations. Some evidence suggests that *ATM* mutations could also be involved in resistance to *CDK4*/*6* inhibitors in luminal positive BC. Relative to the triple-negative BC subtype, in the era of immunotherapy for early and advanced disease, the role of *ATM* mutations in predicting treatment efficacy and good or poor response to immune checkpoint inhibitors, such as PD1/PDL1 inhibitors, could be interesting. As in colon cancer, immunotherapy proved to be effective in patients with alterations of mismatch repair gene alterations; we could hypothesize that *ATM* mutations could enhance the genomic instability of DNA and enhance the immunotherapy response in triple-negative BC patients. *ATM* missense mutations among women diagnosed with BC before the age of 45 years could enhance the risk of contralateral breast cancer in radio-treated patients after a long latency period. In addition, although *ATM* is considered a tumor suppressor, *ATM* mutations can enhance chemo-radioresistance to tumor cells, and this could be useful to explore as a potential target for cancer treatment. In order to overcome the drug resistance in *ATM*-deficient tumors, some studies tested the *ATR*-checkpoint kinase 1 (*Chk1*) cascade as a potential target [74,75,76,77,78], showing an improved response to therapy of these tumors. Based on these findings, additional studies are needed to elucidate the unique characteristics of *ATM*-associated BC, which may have implications on personalized management, from diagnosis to treatment and follow-up of BC patients.

## Data Availability

No new data were created or analyzed in this study. Data sharing is not applicable to this article.
